# Repeated Bilateral Transcranial Direct Current Stimulation over Auditory Cortex for Tinnitus Treatment: A Double-Blinded Randomized Controlled Clinical Trial

**DOI:** 10.3390/brainsci14040373

**Published:** 2024-04-12

**Authors:** Ali Yadollahpour, Samaneh Rashidi, Nader Saki, Pramod Singh Kunwar, Miguel Mayo-Yáñez

**Affiliations:** 1 Department of Psychology, University of Sheffield, Sheffield S1 2LT, UK; yadollahpour.a@gmail.com; 2 Bioelectromagnetic Clinic, Imam Khomeini Hospital, Ahvaz Jundishapur University of Medical Sciences, Ahvaz 61357-15794, Iran; 3 Department of Psychology, University of Surrey, Guildford GU2 7XH, UK; samanehrashidi92@gmail.com; 4 Hearing and Speech Research Center, Ahvaz Jundishapur University of Medical Sciences, Ahvaz 61357-15794, Iran; ahvaz.ent@gmail.com; 5 Department of Pharmaceutics and Pharmacy Practice, School of Pharmacy, Mount Kenya University, Thika P.O. Box 342-01000, Kenya; pkunwar@mku.ac.ke; 6 Department of Otorhinolaryngology—Head and Neck Surgery, Complexo Hospitalario Universitario A Coruña (CHUAC), 15006 A Coruña, Spain; 7 Department of Otorhinolaryngology—Head and Neck Surgery, Hospital San Rafael (HSR), 15006 A Coruña, Spain; 8 Otorhinolaryngology—Head and Neck Surgery Research Group, Institute of Biomedical Research of A Coruña (INIBIC), Complexo Hospitalario Universitario de A Coruña (CHUAC), Universidade da Coruña (UDC), 15006 A Coruña, Spain

**Keywords:** tinnitus, transcranial direct current stimulation, audiology, intractable, auditory cortex

## Abstract

Transcranial direct current stimulation (tDCS) is a non-invasive and painless technique of brain neuromodulation that applies a low-intensity galvanic current to the scalp with the aim of stimulating specific areas of the brain. Preliminary investigations have indicated the potential therapeutic efficacy of multisession tDCS applied to the auditory cortex (AC) in the treatment of chronic tinnitus. The aim of this study was to explore the therapeutic effects of repeated sessions of bilateral tDCS targeting the AC on chronic tinnitus. A double-blinded randomized placebo-controlled trial was conducted on patients (n = 48) with chronic intractable tinnitus (>2 years duration). Participants were randomly allocated to two groups: one receiving tDCS (n = 26), with the anode/cathode placed over the left/right AC, and the other receiving a placebo treatment (n = 22). A 20 min daily session of 2 mA current was administered for five consecutive days per week over two consecutive weeks, employing 35 cm^2^ electrodes. Tinnitus handicap inventory (THI) scores, tinnitus loudness, and tinnitus distress were measured using a visual analogue scale (VAS), and were assessed before intervention, immediately after, and at one-month follow-up. Anodal tDCS significantly reduced THI from 72.93 ± 10.11 score to 46.40 ± 15.36 after the last session and 49.68 ± 14.49 at one-month follow-up in 18 out of 25 participants (*p* < 0.001). The risk ratio (RR) of presenting an improvement of ≥20 points in the THI after the last session was 10.8 in patients treated with tDCS. Statistically significant reductions were observed in distress VAS and loudness VAS (*p* < 0.001). No statistically significant differences in the control group were observed. Variables such as age, gender, duration of tinnitus, laterality of tinnitus, baseline THI scores, and baseline distress and loudness VAS scores did not demonstrate significant correlations with treatment response. Repeated sessions of bilateral AC tDCS may potentially serve as a therapeutic modality for chronic tinnitus.

## 1. Introduction

Tinnitus is an auditory condition characterized by a subjective phantom sound sensation in the absence of external sound. It affects 10–15% of the adult population [1,2]. Tinnitus can present in different forms, including pulsatile, hissing, buzzing, ringing, tones, or a combination of these [2,3]. The main causes of tinnitus include trauma to the auditory periphery, such as hearing loss accompanied by noise, or a lesion to an auditory nerve [1], as well as maladaptive plastic changes in the auditory network induced by damage to the early stages of the auditory pathway [2,4,5]. Although clinically positive symptoms of hearing loss are not a necessary precondition for tinnitus, recent studies have shown that different forms of hearing loss which are not detected by conventional audiometric assessments may have some correlation with tinnitus [6]. However, neuroimaging, neuroanatomy, and evoked potential studies have shown that tinnitus-related anomalies are present not only throughout the auditory system, but also in several non-auditory brain areas [2,7,8,9,10,11]. Recent neuroimaging studies have demonstrated that abnormal activity observed in the primary and secondary auditory cortices could underlie the phantom auditory perception itself; whereas the abnormal activities present in the non-auditory areas associated with cognitive, attentional, and limbic processes could be involved in the unpleasant and distressing aspects of tinnitus [12,13]. Therefore, in any therapeutic or management strategy, tinnitus should be regarded as a multifaceted disorder involving an extensive network comprising multiple overlapping brain areas. Typically, this condition is accompanied by various significant comorbidities like depression, anxiety, and sleep disturbances, rendering it a debilitating condition [1,2,14,15]. Numerous pharmaceutical agents are employed in the treatment of tinnitus; nonetheless, a substantial proportion of patients do not respond to treatment [16].

Different non-pharmaceutical techniques for tinnitus treatment have been developed, such as cognitive behavioral therapies [17], noise-masking modality [5], and neurofeedback [18], but their effectiveness remains limited. Transcranial direct current stimulation (tDCS) is a safe and easy to use form of neuromodulation with potential therapeutic efficacy in different neuropsychiatric disorders [5,19,20,21,22], as well as the capability to enhance different cognitive functions in healthy individuals [23].

Similar to other neuromodulation and neurostimulation approaches, the main rationale for choosing the site of stimulation as well as the electrode montage in tDCS applications for tinnitus is targeting the affected brain areas to modulate either the tinnitus percept or its affective components through disrupting the underlying pathological neural activity. In this regard, given the associations between tinnitus and the structural and functional abnormalities in the dorsolateral prefrontal cortex (DLPFC) [5,10,24] and auditory cortex (AC) [25,26], these two sites were the main targets in previous studies.

In addition to the therapeutic outcomes that have resulted from disturbing tinnitus-induced impaired neural activities, repeated sessions of tDCS have reportedly reduced or increased neural excitability, depending on the polarity of the electrode persisting beyond the termination of tDCS intervention [27,28]. This altered excitability can lead to neuroplasticity with therapeutic effects for tinnitus. Therefore, abnormal excitability in the auditory pathways and maladaptive plastic changes in auditory and limbic cortical areas in tinnitus have led to the idea of treating tinnitus by modulating these abnormalities through single or repeated sessions of tDCS. Using single and repeated sessions of tDCS targeting either the DLPFC or AC have resulted in immediate beneficiary effects in tinnitus patients; however, most of the observed effects were transient and did not translate into long-term improvements [5,10,24,25,26,29]. Different review papers have evaluated the efficacy of tDCS for the treatment of tinnitus [30,31,32,33,34,35]. Initial studies focused on single or few sessions of tDCS over the prefrontal cortex (PFC) [5,24,36] and auditory cortex [25,26,37,38] or left temporal area (LTA) [39,40,41]. Later studies further focused on repeated sessions of tDCS, especially cathodal tDCS [42]. Based on the theoretical and experimental findings of the initial studies, repeated sessions of cathodal tDCS might have therapeutic effects on tinnitus [38,43,44,45,46,47,48]. Accordingly, several studies have investigated the effects of repeated sessions, using longer periods in each session and higher intensities of cathodal tDCS in tinnitus [24,37,45,49]. The main target site was the PFC, particularly the dorsolateral PFC (DLPFC), and the most frequent electrode montage was bifrontal [5,10,50,51]. In bifrontal DLPFC, the electrode montage was either anode left/cathode right DLPFC or anode right/cathode left DLPFC [5,48,52]. Recent studies have used different electrode montages, including high definition (HD) tDCS, for tinnitus treatment and have reported promising though controversial therapeutic effects [53,54,55]. Further studies are needed to reach a definitive conclusion.

Most of the initial tDCS studies assessed the therapeutic efficacy of a single session of tDCS on tinnitus. Later, several studies investigated the effects of repeated sessions of tDCS on tinnitus symptoms, and most of them targeted the DLPFC [24,52]. Similarly, studies with repeated tDCS sessions as a protocol for tinnitus treatment have been conducted targeting the temporal or temporoparietal (auditory) cortex [37,40,47,56]. The results of these studies have been heterogeneous, necessitating further placebo-controlled randomized studies to reach a decisive conclusion. In addition, most of the previous studies have investigated the transient effects of tDCS, and in most cases the after-effect assessments did not extend beyond some hours.

To our knowledge, this is the first double blinded randomized placebo-controlled trial investigating the effect of repeated sessions of bilateral anodal/cathodal tDCS over left/right AC with one month follow-up for the treatment of chronic intractable tinnitus.

## 2. Materials and Methods

### 2.1. Patients

Consecutive patient recruitment was conducted at the clinics of the Tinnitus Clinic at the Khuzestan Cochlear Implant Center (Ahvaz, Iran). The inclusion criteria were idiopathic chronic and medications resistant tinnitus (THI ≥ 38) with disease duration of more than 2 years [16], age range of 18 to 65 years old, and no use of medications or sound therapy at the time of intervention. For all patients a wash-out period of 3 months was applied. The choice of a THI score greater than or equal to 38 was based on the recommendations of the British Association of Otolaryngologists, Head and Neck Surgeons. With this score, patients with moderate, severe, and catastrophic tinnitus were included [57].

The exclusion criteria were a history of epileptic seizures, brain trauma, severe psychotic and psychiatric disorders, concurrent severe vertigo, Meniere’s disease, severe organic comorbidity, using a pacemaker or defibrillator, a present pregnancy, neurologic disorders such as brain tumors, and individuals being treated for mental disorders.

All prospective subjects underwent complete audiometric and neurologic examinations by experienced specialists. This clinical trial was a part of long-term study designed to comprehensively investigate the efficacy of different tDCS protocols for treatment of chronic and refractory tinnitus by the bioelectromagnetic clinic of Imam Hospital, Ahvaz, Iran.

### 2.2. Design

**Figure 1:**
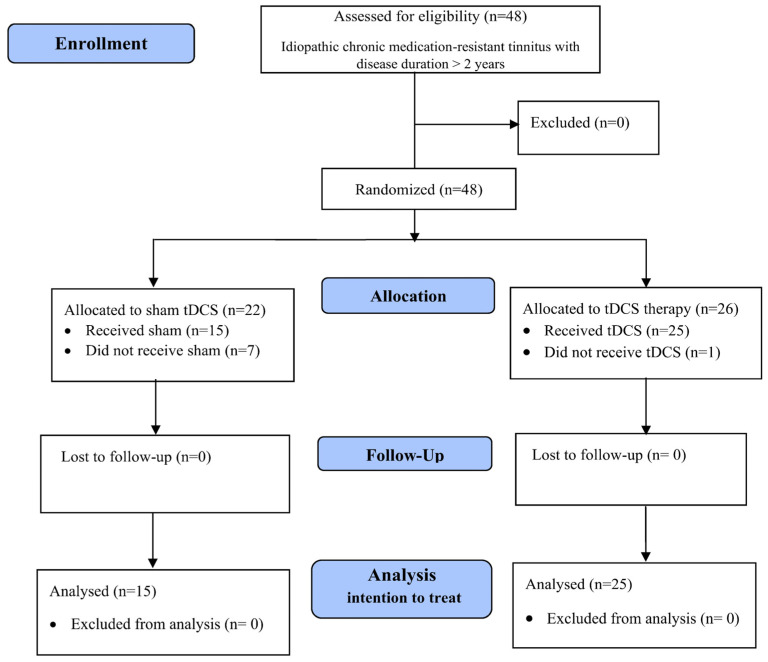
Diagram flow of trial.

This study was designed as a double-blind randomized controlled clinical trial with the aim of investigating the therapeutic effectiveness of administering repeated sessions of anodal tDCS over the AC, totaling 10 sessions, in the context of chronic and intractable tinnitus (see Figure 1). Following the application of predefined inclusion and exclusion criteria, 48 patients were included in the study. These patients were randomly allocated to two groups: one receiving tDCS (n = 26), with the anode/cathode placed over the left/right AC, and another receiving a placebo treatment (n = 22). The patients were randomly allocated to the two groups; the randomization was performed using a simple randomization process. The two groups were carefully matched in terms of age, gender, and ethnicity. To mitigate potential subjective biases, a comprehensive blinding procedure was implemented. This involved ensuring that patients, the researchers responsible for evaluating outcomes during the experiments and follow-up period, as well as the researcher conducting the statistical analyses, were all unaware of the specific treatment protocol to which each patient was assigned. Prior to enrollment, patients received a clear and detailed explanation of the study’s objectives, potential benefits, and possible side effects.

### 2.3. Transcranial Direct Current Stimulation Protocol

tDCS was administered using a pair of surface sponges (35 cm^2^) soaked in saline solution and delivered via a specially developed battery-powered constant current stimulator capable of a maximum output of 4 mA. The tDCS device utilized in this study was the OASIS Pro^TM^ device manufactured by Mind Alive Inc. (Edmonton, AB, Canada).

The tDCS protocol involved a 2 mA current administered daily for 20 min over five consecutive days per week, for a duration of two consecutive weeks. In the anodal tDCS condition, the anode was positioned over the left auditory cortex (midway between T3 and F7), while the cathode was placed over the right auditory cortex (midway between T4 and F8), using 35 cm^2^ electrodes. The site for stimulation was determined by the International 10–20 Electroencephalogram system, where the left and right AC corresponded to halfway T3–F7 and halfway T4–F8, respectively [58]. According to the tDCS specifications, in both cases, intervention and placebo, the DC current was initially increased in a ramp-like fashion for about 10 s until it reached 2 mA. In the placebo tDCS, the electrode montage was the same as in real tDCS, except that the device was turned off 40–45 s after the start of the session without the patient being aware of this fact. These parameters for placebo stimulation were chosen based on previous reports that the perceived sensations on the skin, such as tingling, usually disappear in the first 30 s after tDCS activation [59,60].

### 2.4. Evaluations

The tinnitus quality for each patient was determined through a clinical interview by a tinnitus expert otolaryngologist, being categorized as: buzzing, cicadas, high pitch whistling, hissing, humming, ringing, pulsating, thumping, and/or ticking. The class of hearing loss in both ears was assessed, based on the World Health Organization criteria, as normal hearing threshold (<20 dB), mild hearing loss (20–40 dB), moderate hearing loss (41–70 dB), severe hearing loss (70–90 dB), and profound hearing loss (>90 dB). The tinnitus laterality and evolution time were also determined. Pure-tone audiometry was performed using an AC 40 dual channel Audiometer (Interacoustics Co., Middelfart, Denmark). The hearing thresholds were recorded over the frequency ranges of 250 to 8000 Hz for air conduction and 500 to 4000 Hz for bone conduction pathways, using the modified Hughson–Westlake Method as recommended by the American National Standard Institute ANSI S3.6 (American National Standard Institute, 1996). Pure-tone audiometry was considered normal when the hearing thresholds at all frequencies were below 20 dBHL.

Tinnitus handicap inventory (THI) score was assessed prior to intervention, and then immediately after, 1 h after, and 1 month after the last stimulation [61]. Tinnitus loudness and distress were assessed using a numeric visual analog 0–10 rating scale before intervention, and immediately, one hour, one week, and one month after last stimulation. After the completion of the intervention in the placebo-tDCS, the blinding quality of the study was assessed through asking each patient to determine which type of stimulation they had received.

### 2.5. Statistical Analysis

The analysis was conducted on an intention-to-treat basis. Statistical analyses were performed using Stata Version 14.2 (StataCorp LLC., College Station, TX, USA). Qualitative variables were expressed as a percentage. Continuous variables were expressed as a mean, median (minimum, maximum), and standard deviation (SD). Baseline characteristics of patients were compared between the two groups using an independent samples student’s *t*-test for continuous variables, and a Chi-square test for qualitative variables. Otherwise, Mann-Whitney’s test was used with no significant Levene’s test variables. Univariate and multivariate logistic regression tests were used to identify a possible association between the basal variables and the efficacy of the therapy, considering this as a decrease in ≥20 points between the value of THI pre-intervention versus post-intervention. Statistical significance was determined at *p* < 0.05. Interaction terms inclusion was evaluated by a chunk test based on the likelihood ratio test with a significance level <0.05. The presence of possible confounding variables was evaluated, adjusting the model if the changes were >10%.

## 3. Results

### 3.1. Participants

Forty-eight patients (F = 26, M = 22; mean age: 48.67 ± 7.81 years) who met the inclusion criteria of the study were consecutively evaluated for eligibility and entered the study. The patients were randomly divided into two groups of real tDCS (n = 26) and placebo tDCS (n = 22) using simple randomization. One patient from the real tDCS group and seven patients from the placebo tDCS group did not finish the sessions or start the intervention. Twenty-five patients from the real tDCS group (F = 14, M = 11; mean age of 47.52 ± 7.51 years; tinnitus duration time of 7.48 ± 3.99 years) and 15 patients from the placebo tDCS group (F = 8, M = 7; mean age of 47.67 ± 7.96 years; tinnitus duration time of 7.60 ± 3.60 years) finished the study and were considered in the analysis. The evaluation of hearing loss based on laterality is detailed in Table 1 and Supplementary Tables S1 and S2.

The distribution of tinnitus localization was 21 (52.5%) cases on the right side, 16 (40%) on the left side, and 3 (7.5%) bilateral. The distribution of tinnitus quality is found in Table 2.

The baseline values of THI, tinnitus loudness, and tinnitus distress are found in Table 3. Sociodemographic and basal variables did not show statistically significant differences between the tDCS group and the placebo group for age (*p* = 0.954), basal THI value (*p* = 0.629), loudness (*p* = 0.708) VAS, or distress VAS (*p* = 0.971) (Table 3). The repeated sessions of tDCS were well-tolerated by all patients; there were no adverse effects reported, and all patients completed the study. No follow-up losses were recorded during the study period.

### 3.2. Response Variables

**Figure 2:**
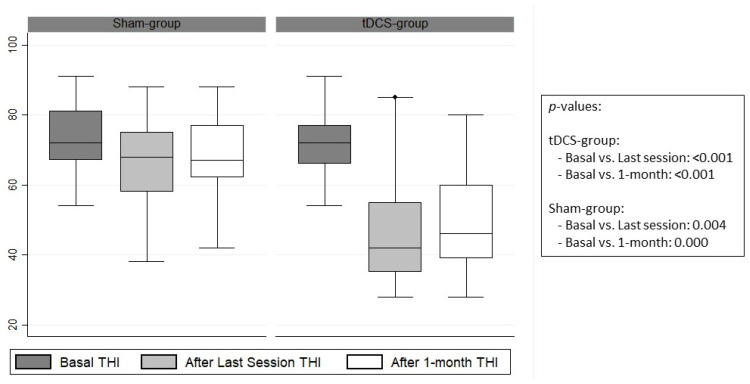
Comparative diagram of THI scores between groups before and after treatment.

Statistically significant differences were found in the response variables. The mean post-intervention THI was 46.40 ± 15.36 for the tDCS group and 66.73 ± 14.30 for the placebo group (*p* < 0.001). These significant differences continued to be present one month after the last tDCS session, with values of 49.68 ± 14.49 for the tDCS group and 66.73 ± 11.97 for the placebo group (*p* < 0.001) (Figure 2). Differences were also found in loudness and distress VAS collected immediately after (*p* = 0.008), after an hour (*p* = 0.001), after a week (*p* = 0.001), and after a month (*p* = 0.003) following the last tDCS session (Table 3).

The comparison of basal and response variables according to the groups showed that in the tDCS group, there were significant differences comparing pre-intervention data with immediate post-intervention distress VAS and loudness VAS variables (*p* < 0.001), between the basal THI and immediate after last session THI with a *p* < 0.001 (CI 95%, 18.66–31.10), and between the basal THI with after 1 month THI with *p* < 0.001 (CI 95%, 15.96–27.24). In addition, significant differences were also found between the immediate after last session THI and after 1 month THI (*p* = 0.011; CI 95%, −5.75–−0.81).

On the other hand, in the placebo group, no significant differences were found between basal VAS for loudness and after an hour following the last session (*p* = 0.096; CI 95%, −0.07–0.73), after 1 week following the last session (*p* = 0.334; CI 95%, −0.08–0.21), and after 1 month following the last session (*p* = 0.337; CI 95%, −0.24–0.09). The same pattern occurred with basal VAS for distress and after an hour following the last session (*p* = 0.104; CI 95%, −0.06–0.60), after 1 week following the last session (*p* = 0.271; CI 95%, −0.17–0.57), and after 1 month following the last session (*p* = 0.164; CI 95%, −0.33–0.06). In addition, no significant differences were found between THI immediately following the last session and THI after 1 month following the most recent intervention (*p* = 1; CI 95%, −4.00–4.00). Interestingly, in this group, statistically significant differences were found between the basal THI value and the immediate post-intervention value (*p* = 0.004; CI 95%, 2.32–10.08), the basal THI and the 1 month post-intervention THI value (*p* = 0.000; CI 95%, 4.08–8.32), the immediate loudness VAS (*p* = 0.007; CI 95%, 0.21–1.12), and the immediate distress VAS (*p* = 0.007; CI 95%, 0.19–1.01).

One participant of 15 in the placebo group and 18 of 25 in the tDCS-group underwent an improvement of 20 or more points in THI score immediately after the last stimulation. This improvement was not maintained in the placebo group at the one-month follow-up, whereas in the tDCS group, the improvements persisted for one month in 14 of the subjects (Table 4).

### 3.3. Multivariate Analysis

Considering a reduction of 20 points or more in THI score as the positive treatment response, a logistic regression was performed with the THI values immediately after the last session (Table 5) and with THI values after one month (Table 6) as a dependent variable. The variables selected in the final model, after removing potential confounding factors, were sex, age, tinnitus duration since its onset, basal THI, basal VAS loudness, and basal VAS distress.

No statistically significant differences were found in the possible effect of baseline variables on response to treatment, with the exception of the tinnitus duration since its onset. In the univariate model, a possible negative association between the evolution time of tinnitus and the positive response to treatment after the last session (OR = 0.71; *p* = 0.019) and after one month (OR = 0.73; *p* = 0.028) was observed. This significance was lost in the multivariate model in the first case and maintained in the second (OR = 0.60; *p* = 0.027).

## 4. Discussion

In this study, we evaluated the safety and effectiveness of repeated sessions of tDCS with a combined stimulation approach. Specifically, we conducted 10 tDCS sessions targeting the auditory cortex (AC), with the anode placed over the left AC and the cathode over the right AC, in an effort to alleviate tinnitus symptoms.

Our primary findings indicate that this tDCS protocol had a positive impact on tinnitus symptoms, leading to significant reductions in THI scores, as well as in the VAS assessments of tinnitus loudness and distress. These assessments were conducted immediately following the completion of the last session, as well as at one week and one month after the treatment. It is important to note that comparing these results with previous studies is challenging due to the unique nature of our research. While there have been open trials and randomized controlled tDCS studies targeting either the left temporoparietal area (LTA)/AC [5,26,39,40,56,62,63] or the PFC [10,24,37,51,52,64,65], to the best of our knowledge, this is the first randomized, double-blind, parallel, placebo-controlled tDCS study to employ this specific protocol, including a one-month follow-up. Our approach involved bilateral anodal/cathodal tDCS over the left/right AC. A review of the existing literature reveals only one similar protocol used by Vanneste et al., in which they applied 1.5 mA bilaterally to the AC (T3 + T4), but no statistically significant differences were found in their results [65]. The statistically significant variations in THI found in the placebo group should be taken with caution. One of the strengths of the analysis conducted is to establish a point considered as clinical improvement (a decrease of >20 in THI). Not every statistically significant change translates into a clinically significant change (Figure 2). In fact, Zeeman et al. established a criterion of at least seven points in THI for significant clinical improvement, a value below the one required in our study [66].

Tinnitus research presents a challenge due to the subjective nature of the condition. Determining a clinically significant change in tinnitus is not feasible through objective measures, necessitating reliance on questionnaires by investigators. We chose to utilize the THI as our primary outcome measure, following recommendations from the Tinnitus Research Initiative [67]. While the THI served as our primary outcome measure, we also incorporated a VAS to assess both tinnitus loudness and distress, a methodology employed in previous studies [64]. A reduction in the VAS for distress may suggest an emotional improvement stemming from participation in the study. However, unlike previous interventions, this time the effect was both statistically significant and enduring over time. In spite of these positive results, data should have been collected in reference to the possible comorbidities suffered by the participants (anxiety, depression …) because they may be factors that influenced the outcome. Our positive results are in agreement with those from other studies using VAS as primary outcome measure [10,39,52,68].

We conducted daily 20 min sessions, five days a week, for two consecutive weeks. Previous tDCS studies have noted temporary reductions in tinnitus following stimulation of the AC or PFC, but these effects displayed significant inter-individual variability and were often limited in duration [10,24,26,39,40,51,56,62,64,65,68]. The reductions observed in this study were comparable to those in previous research, with the distinction that they exhibited a more prolonged duration. This contrasts with the findings from multisession tDCS studies; none of them identified long-term reductions in tinnitus severity over time [24,26,39,40,52,56,64]. There is evidence suggesting cumulative effects from repeating interventions, as demonstrated in prior studies [24,52]. However, what distinguishes tDCS in our study is the apparent longevity of its effects. It would be intriguing to investigate whether these effects can be maintained over time through multisession visits at intervals. The maintenance sessions treatment modality has previously been successfully implemented in cases of depression or chronic pain [69,70]. This would provide valuable insights into the extent of improvement participants experience over time without additional sessions, ultimately helping us determine the optimal number of sessions required to alleviate tinnitus and the true duration of the effect.

The results of logistic regression analyses revealed that tinnitus duration since onset as a variable had a significant relationship with the response to tDCS therapy, and this significant effect was statistically significant both immediately after the last session and one month after the last session. This relationship indicates that patients with longer durations of tinnitus were less responsive to the treatment in a way that is clinically significant. This finding could pave the way for developing clinical guidelines to identify the candidacy of patients for tDCS treatment. However, this factor should be considered along with other factors that have significant effects on treatment response. Conducting further studies with larger sample sizes is needed to determine the relationship between tinnitus onset duration and treatment response.

The suitability of patients for clinical intervention is a very important clinical point to make as it provides guidance on candidacy for tDCS.

The concept of maladaptive plasticity suggests that therapeutic stimulation might require an extended duration to induce its intended effects. Following this idea, and based on the existing literature, it would be interesting in the future to combine some drugs that favor neuronal plasticity with tDCS, thus enhancing the benefits through a synergistic effect. One such drug could be Acamprosate; a double-blinded placebo-controlled crossover study has demonstrated its positive effects on tinnitus perception [71]. Specifically, during the active drug period, there was a decrease in tinnitus measures, whereas there was no notable change in these measures during the placebo period.

One limitation of this study to highlight was the non-collection of data relating to changes in auditory threshold and speech perception in order to assess both the safety of tDCS and the possibility of this influencing response to treatment. Regarding the first point, an evidence-based update of this topic has recently been published, confirming the complete safety of the procedure [28]. Consistent with the argument that hearing loss negatively predicts the outcome of stimulation treatment, two recent studies have demonstrated that improvements in tinnitus resulting from multisession tDCS, when combined with either the use of hearing aids [40] or customized notched-music training [56], appear to be unrelated to the number of tDCS sessions administered. Another limitation of this study was that we did not include the variables of quality of tinnitus and hearing loss in the multivariate statistical analysis. In our study, there was no matching based on hearing loss, nor the use of hearing aids. Conducting controlled clinical trials with larger sample sizes is necessary to avoid potential bias. Considering the various types of tinnitus, including ringing, pulsatile, and unilateral versus bilateral, quality of tinnitus as a variable holds potential value and warrants further exploration through controlled studies with larger sample sizes. Additionally, incorporating objective modalities such as neuroimaging and audiometric assessments could be crucial in developing effective individualized treatment plans. Finally, another limitation of our study was the short follow-up period (1 month) for outcome evaluation. An assessment at 6 or 12 months is necessary in the future to confirm the duration of the effect and the need for further tDCS sessions.

The absence of standardized study methodology presents an additional challenge in tinnitus research. The substantial diversity in design options, such as tDCS, tACS, and tRNS, electrode placements, and stimulation parameters, creates a multitude of potential neuromodulation approaches, which could result in a disorganized collection of data. The therapeutic effectiveness of tDCS remains uncertain until data from larger multicenter randomized controlled trials become available. To address this issue, a systematic approach is essential, accompanied by well-defined methods and outcome measures. The “Evidence-based guidelines on the therapeutic use of transcranial direct current stimulation” concludes that there is a level of evidence in favor of the probable absence of efficacy of anodal tDCS of the left temporo-parietal area to relieve chronic tinnitus [44]. In this scenario, the results of this study are more relevant due to their statistical and clinical significance, the design of the trial, as well as the new placement of the electrodes, opening a new line of research in this regard.

## 5. Conclusions

This study represents the inaugural double-blinded randomized placebo-controlled trial that delves into the impact of repeated sessions of bilateral anodal/cathodal tDCS over the left/right auditory cortex (AC) with a one month follow-up, aimed at treating chronic intractable tinnitus. The results demonstrate the therapeutic potential of multisession tDCS in alleviating tinnitus symptoms, reducing intensity and distress, and notably, these therapeutic effects persisted for up to one month after intervention. The repeated application of bilateral tDCS appears to hold promise as a potential therapeutic approach for chronic tinnitus. However, further controlled studies are essential to arrive at a definitive conclusion.

## Figures and Tables

**Table 1:** Distribution of hearing loss.

			TDCS-Group	Placebo-Group	Total
Right ear	Normal	N	10	5	15
		%	40	33.3	37.5
	Mild	N	7	5	12
		%	28	33.3	30
	Moderate	N	5	4	9
		%	20	26.7	22.5
	Profound	N	3	1	4
		%	12	6.7	10
Left ear	Normal	N	11	5	16
		%	44	33.3	40
	Mild	N	10	6	16
		%	40	40	40
	Moderate	N	3	3	6
		%	12	20	15
	Profound	N	1	1	2
		%	4	6.7	5

**Table 2:** Distribution of tinnitus quality.

	N	%
Ringing	12	30
Buzzing	2	5
Hissing	7	17.5
Ticking	2	5
High pitch whistling	5	12
Thumping	2	5
Cicadas	5	12.5
Pulsating	5	12.5

**Table 3:** Comparison of the demographic and response variables of the study between the tDCS group and placebo group. The *p*-values in bold indicate statistical significance.

	Mean ± SD		*p*-Value
	tDCS Group	Placebo Group	
Age	47.52 ± 7.51	47.67 ± 7.96	0.954
Tinnitus duration since its onset	7.48 ± 3.99	7.60 ± 3.60	0.924
Basal THI	72.93 ± 10.11	71.90 ± 10.30	0.629
THI post-intervention	46.40 ± 15.36	66.73 ± 14.30	**<0.001**
THI 1-month post-intervention	49.68 ± 14.49	66.73 ± 11.97	**<0.001**
Basal Loudness VAS	7.36 ± 0.81	7.47 ± 0.91	0.708
Loudness VAS immediate after last session	5.60 ± 1.78	6.80 ± 1.52	**0.036**
Loudness VAS 1-h post-last session	5.56 ± 1.78	7.13 ± 1.40	**0.006**
Loudness VAS 1-week post-last session	5.68 ± 1.57	7.40 ± 1.05	**0.001**
Loudness VAS 1-month post-last session	6.64 ± 1.18	7.69 ± 0.85	**0.010**
Basal Distress VAS	7.68 ± 0.55	7.67 ± 0.61	0.971
Distress VAS immediate after last session	5.92 ± 1.25	7.07 ± 1.22	**0.008**
Distress VAS 1-h post-last session	5.92 ± 1.25	7.40 ± 1.12	**0.001**
Distress VAS 1-week post-last session	6.16 ± 1.24	7.47 ± 0.83	**0.001**
Distress VAS 1-month post-last session	6.92 ± 0.99	7.80 ± 0.56	**0.003**

Abbreviations: SD, standard deviation. tDCS, transcranial direct current stimulation. THI, tinnitus handicap index. VAS, visual analog scale.

**Table 4:** Calculation of the risk ratio immediately after the last session and at one month. Note that those with tDCS treatment had a 980% increase in the risk of improving their THI after the last session. After 1 month, no patient in the placebo group showed improvement.

		Improvement of ≥20 Points in the THI			
		Yes	No	Total	Risk Ratio
Immediately after	tDCS treatment	18	7	25	10.8 (IC 95%, 1.6–72.88)
	Placebo group	1	14	15	
	Total	19	21	40	
1-month after	tDCS treatment	14	11	25	2.36 (IC 95%, 1.51–3.7)
	Placebo group	0	15	15	
	Total	14	26	40	

**Table 5:** Results of univariate and multivariate (logistic regression) analysis on possible relationship between the basal variables and the response to tDCS therapy immediately after last session. The *p*-values in bold indicate statistical significance.

		Improvement ≥ 20 in THI Immediate after Last Session					
		Yes (N = 18)	No (N = 7)	Univariate Model		Multivariate Model	
				*p*-Value	OR (IC 95%)	*p*-Value	OR Adjusted (IC 95%)
Sex	Male	7 (38.89)	4 (57.14)	0.413	0.48 (0.08–2.81)	0.156	0.06 (0.00–2.89)
	Female	11 (61.11)	3 (42.86)	0.413	2.10 (0.36–12.32)	0.156	16.33 (0.35–771.31)
		**Mean ± SD**	**Mean ± SD**	**Univariate Model**		**Multivariate Model**	
				***p*-Value**	**OR (IC 95%)**	***p*-Value**	**OR Adjusted (IC 95%)**
Age		46.72 ± 7.68	49.57 ± 7.21	0.392	0.95 (0.84–1.07)	0.325	0.325 (0.70–1.12)
Tinnitus duration since its onset		6.17 ± 3.40	10.86 ± 3.53	**0.019**	0.71 (0.53–0.95)	0.064	0.64 (0.39–1.03)
Basal THI		70.94 ± 11.62	72.14 ± 7.95	0.795	0.99 (0.91–1.08)	0.900	1.01 (0.88–1.16)
Basal VAS Loudness		7.17 ± 0.71	7.86 ± 0.90	0.071	0.29 (0.08–1.11)	0.166	0.14 (0.01–2.26)
Basal VAS Distress		7.71 ± 6.49	7.67 ± 0.59	0.845	0.85 (0.16–4.43)	0.556	3.36 (0.06–188.8)

Abbreviations: OR, odds ratio. SD, standard deviation. THI, tinnitus handicap index. VAS, visual analog scale.

**Table 6:** Results of univariate and multivariate (logistic regression) analysis on possible relationship between the basal variables and the response to tDCS therapy 1 month after last session. The *p*-values in bold indicate statistical significance.

		Improvement ≥ 20 in THI after 1-Month					
		Yes (N = 13)	No (N = 12)	Univariate Model		Multivariate Model	
				*p*-Value	OR (IC 95%)	*p*-Value	OR Adjusted (IC 95%)
Sex	Male	4 (30.77)	7 (58.33)	0.171	0.32 (0.06–1.64)	0.093	0.09 (0.01–1.50)
	Female	9 (69.23)	5 (41.67)	0.221	3.15 (0.61–16.31)	0.093	11.17 (0.67–187.07)
		**Mean ± SD**	**Mean ± SD**	**Univariate Model**		**Multivariate Model**	
				***p*-Value**	**OR (IC 95%)**	***p*-Value**	**OR Adjusted (IC 95%)**
Age		45.85 ± 8.63	49.34 ± 5.91	0.249	0.93 (0.83–1.05)	0.694	0.97 (0.82–1.14)
Tinnitus duration since its onset		5.62 ± 2.99	9.5 ± 4.06	**0.028**	0.73 (0.55–0.97)	**0.027**	0.60 (0.38–0.94)
Basal THI		72.54 ± 11.11	69.92 ± 10.26	0.529	1.03 (0.95–1.11)	0.224	1.10 (0.94–1.28)
Basal VAS Loudness		7.15 ± 0.69	7.58 ± 0.90	0.191	0.48 (0.16–1.44)	0.819	0.82 (0.16–4.30)
Basal VAS Distress		7.54 ± 0.66	7.83 ± 0.39	0.199	0.32 (0.06–1.81)	0.120	0.08 (0.00–1.95)

Abbreviations: OR, odds ratio. SD, standard deviation. THI, tinnitus handicap index. VAS, visual analog scale.

## Data Availability

The data used in this study are available upon request from the corresponding author due to their containing information that could compromise the privacy of the study participantsants.

## Supplementary Materials

The following supporting information can be downloaded at: https://www.mdpi.com/article/10.3390/brainsci14040373/s1, Table S1: Participants’ Characteristics of tDCS intervention. Table S2. Participants’ characteristics of sham stimulation.
