# Supplementary material for: Repeated Bilateral Transcranial Direct Current Stimulation over Auditory Cortex for Tinnitus Treatment: A Double-Blinded Randomized Controlled Clinical Trial
Source: Brain Sci. 2024 Apr 12;14(4):373. doi: 10.3390/brainsci14040373 (PMC11048041; doi:10.3390/brainsci14040373)
Supplement: Supplementary file 1 [file brainsci-14-00373-s001.zip › brainsci-2581998-supplementary.pdf]

Supplementary Table S1. Participants' Characteristics of tDCS intervention.

Patient No.	Age (Years)	Sex	Hearing Loss Right/Left
1	55	F	M/P
2	43	F	N/L
3	47	F	P/L
4	63	F	M/L
5	48	F	N/N
6	42	F	N/N
7	38	F	N/N
8	54	F	N/L
9	55	F	M/L
10	33	F	N/N
11	48	F	L/L
12	43	F	M/M
13	42	F	L/N
14	47	F	M/L
15	57	M	L/M
16	47	M	L/N
17	48	M	L/N
18	38	M	N/N
19	48	M	N/N
20	52	M	N/N
21	62	M	N/L
22	45	M	L/L
23	50	M	P/L
24	36	M	P/M
25	47	M	M/N

Class of hearing loss: N, normal hearing threshold (<20 dB); L, mild hearing loss (20–40 dB); M, moderate hearing loss (41–70 dB); S, severe hearing loss (70–90 dB); P, profound hearing loss (>90 dB).

Supplementary Table S2. Participants' characteristics of sham stimulation.

Patient No.	Age (Years)	Sex	Hearing Loss Right/Left
1	55	F	M/P
2	43	F	N/L
3	47	F	P/L
4	63	F	L/M
5	48	F	N/L
6	42	F	N/N
7	38	F	N/N
8	54	F	L/N
9	55	M	M/L
10	33	M	N/N
11	48	M	L/L
12	43	M	M/M
13	42	M	L/N
14	47	M	M/L
15	57	M	L/M

Class of hearing loss: N, normal hearing threshold (<20 dB); L, mild hearing loss (20–40 dB); M, moderate hearing loss (41–70 dB); S, severe hearing loss (70–90 dB); P, profound hearing loss (>90 dB).
